# Increased circulating β_2_-adrenergic receptor autoantibodies are associated with smoking-related emphysema

**DOI:** 10.1038/srep43962

**Published:** 2017-03-06

**Authors:** Jia-yi Hu, Bei-bei Liu, Yi-peng Du, Yuan Zhang, Yi-wei Zhang, You-yi Zhang, Ming Xu, Bei He

**Affiliations:** 1 Department of Respiratory Medicine, Peking University Third Hospital, Beijing, China; 2 Department of Cardiology, Institute of Vascular Medicine, Peking University Third Hospital, Key Laboratory of Molecular Cardiovascular Sciences, Ministry of Education, Key Laboratory of Cardiovascular Molecular Biology and Regulatory Peptides, Ministry of Health; Beijing Key Laboratory of cardiovascular Receptors Research, Beijing, China

## Abstract

Smoking is a dominant risk factor for chronic obstructive pulmonary disease (COPD) and emphysema, but not every smoker develops emphysema. Immune responses in smokers vary. Some autoantibodies have been shown to contribute to the development of emphysema in smokers. β_2_-adrenergic receptors (β_2_-ARs) are important targets in COPD therapy. β_2_-adrenergic receptor autoantibodies (β_2_-AAbs), which may directly affect β_2_-ARs, were shown to be increased in rats with passive-smoking-induced emphysema in our current preliminary studies. Using cigarette-smoke exposure (CS-exposure) and active-immune (via injections of β_2_-AR second extracellular loop peptides) rat models, we found that CS-exposed rats showed higher serum β_2_-AAb levels than control rats before alveolar airspaces became enlarged. Active-immune rats showed increased serum β_2_-AAb levels, and exhibited alveolar airspace destruction. CS-exposed-active-immune treated rats showed more extensive alveolar airspace destruction than rats undergoing CS-exposure alone. In our current clinical studies, we showed that plasma β_2_-AAb levels were positively correlated with the RV/TLC (residual volume/total lung capacity) ratio (*r* = 0.455, *p* < 0.001) and RV%pred (residual volume/residual volume predicted percentage, *r* = 0.454, *p* < 0.001) in 50 smokers; smokers with higher plasma β_2_-AAb levels exhibited worse alveolar airspace destruction. We suggest that increased circulating β_2_-AAbs are associated with smoking-related emphysema.

Chronic obstructive pulmonary disease (COPD) remains a leading cause of morbidity and mortality worldwide, and tobacco smoking is the dominant risk factor for COPD. Emphysema is defined pathologically as an abnormal, permanent enlargement of the airspaces distal to the terminal bronchioles. In addition, there are still no effective ways to reverse the progress of emphysema. The Evaluation of COPD Longitudinally to Identify Predictive Surrogate End-points (ECLIPSE) cohort study used a cluster analysis to show that COPD patients with severe emphysema underwent a greater reduction in health status, a faster progression of emphysema, a higher exacerbation rate, and a greater deterioration of clinical symptoms^1^. However, only 15–20% of smokers develop COPD and not all COPD patients or smokers exhibit emphysema^2^. This indicates that individual susceptibility to smoke exposure varies among different people, and research on the underlying mechanism may contribute to developing individualized treatments.

Recent evidence suggests that autoimmunity plays a role in the pathogenesis of COPD and emphysema^3^,^4^. The damage-repair process in the development of COPD and emphysema may activate the immune system, resulting in autoantibody production^5^. Previous studies have demonstrated the existence of anti-elastin and anti-endothelial cell (EC) antibodies associated with emphysema, which can cause lung parenchyma destruction and alveolar airspace enlargement, and the levels of these autoantibodies vary among different people^6^,^7^. An autoantigen array analysis found that patients with emphysema produced higher antibody titres and were reactive to an increased number of antigens. Strikingly, the levels of autoantibody reactivity observed in cases of emphysema were increased, even over those detected in rheumatoid arthritis patients and were similar to those associated with lupus^8^.

β_2_-adrenergic receptors (β_2_-ARs) are important targets in the treatment of lung disease. β_2_-adrenergic receptors are widely distributed in lung cells, such as airway smooth muscle cells, epithelial cells, and inflammatory cells^9^,^10^,^11^. β_2_-adrenergic receptor agonists are involved in regulating airway smooth muscle tone^12^. Our previous studies also reported that β_2_-adrenergic receptors are involved in anti-inflammatory and anti-hypersecretory functions *in vivo* and *in vitro*^13^,^14^. Increasing evidence suggests that β-adrenergic receptor (β-AR) autoantibodies act as pathogenic drivers, and the extracellular domains of receptor proteins are targets for autoimmune recognition^15^. However, only a few studies have focused on β_2_-AR autoantibodies (β_2_-AAbs). One type of inhibitory β_2_-AAb against the third extracellular loop of the receptor has been reported to contribute to the adrenergic hyporesponsiveness observed in asthma patients^16^. Agonistic β_2_-AAbs against the second extracellular loop of the receptor have been reported in Chagas’ cardiomyopathy, open-angle glaucoma, and regional pain syndrome^17^. Agonistic β_2_-AAbs against the first extracellular loop of the receptor have been reported in association with Alzheimer’s disease and vascular dementia^18^ and may cause vascular damage. No studies have shown any evidence for a link between emphysema and β_2_-AAbs.

Long-term passive smoking is an effective way to induce emphysema in rats^19^. Our current preliminary studies showed that serum levels of β_2_-AAbs against the β_2_-AR second extracellular loop peptide (ECL_II_ peptide), were increased in passive-smoking rats with emphysema relative to levels in control rats. Thus, we hypothesized that increased levels of β_2_-AAbs may be associated with an increase in the extent of emphysema. To test this hypothesis, we established a rat model of cigarette-smoke exposure (CS-exposure) and a rat active-immune model using β_2_-AR ECL_II_ peptide injections, and we combined CS-exposure and immune activation in another group of rats to explore the changes in serum β_2_-AAb levels and the associated pathological and functional changes in the lungs. We also performed a clinical study in a group of smokers to further study this relationship. The aim of this study was to investigate whether β_2_-AAbs are involved in emphysema.

## Results

### Measurements for the CS-exposure rat model

#### CS-exposed rats showed increased serum β_2_-AAb levels before alveolar airspaces became enlarged

**Figure 1:**
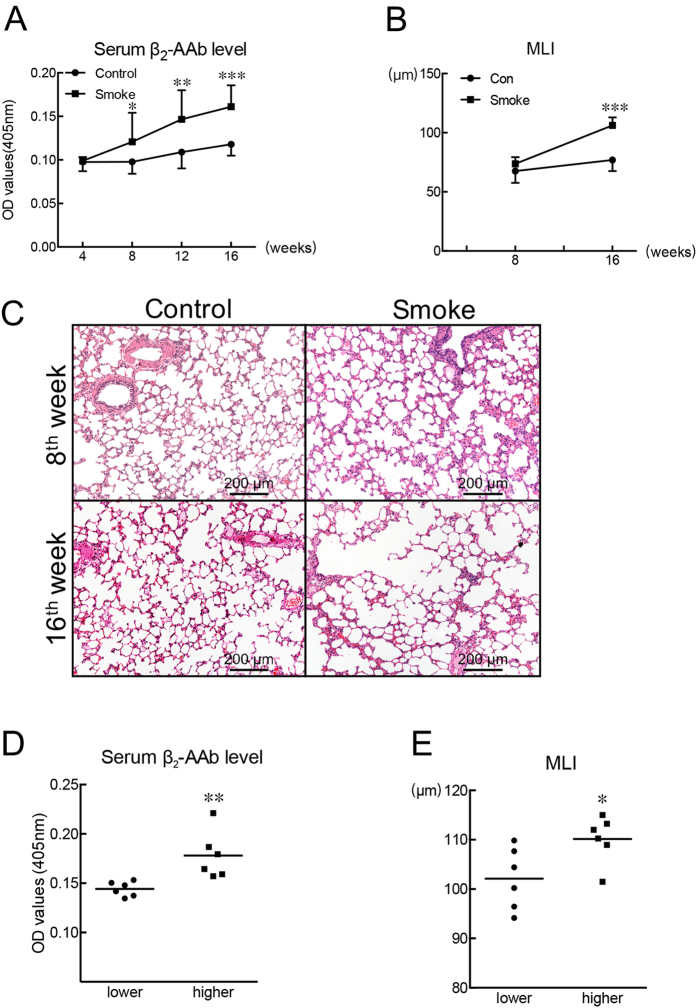
Passive-smoking rats showed higher serum β_2_-AAb levels than control rats before alveolar airspaces became enlarged. (**A**) Levels of β_2_-AAb in serum samples of CS-exposed rats and control rats were assessed at different time points. SE-ELISAs were performed after rats were exposed to clean air or cigarette smoke for 4, 8, 12, or 16 weeks. (**B**) Statistical analyses of mean linear intercept (MLI) measurements of lung sections after rats were exposed to clean air or cigarette smoke for 8 or 16 weeks. (**C**) Sections of lungs from a control rat and from a CS-exposed rat sacrificed at the end of the 8^th^ or 16^th^ week. Haematoxylin-eosin staining, scale bar = 200 μm. (**D**) and (**E**) Lower and higher groups refer to CS-exposed rats with relatively lower and higher serum β_2_-AAb levels, respectively, and results of the statistical analysis of serum β_2_-AAb levels and MLI values of the two groups were shown in panel (D) and (E) respectively. *p < 0.05, **p < 0.01, ***p < 0.001; Control group vs Smoke group or Lower group vs Higher group (n = 18 for the Control group and Smoke group at the 4^th^ or 8^th^ week in panel A, n = 12 for the Control group and Smoke group at the 12 ^th^ or 16^th^ week in panel A; n = 6 for the Control group and Smoke group at the 8^th^ week in panel B, n = 12 for the Control group and Smoke group at the 16^th^ week in panel B; n = 6 for the Lower group and Higher group at the 16^th^ week in panels D and E).

The serum β_2_-AAb levels of CS-exposed rats significantly increased after 8 weeks of CS-exposure, and the significant increase was maintained as CS-exposure proceeded to 16 weeks (Fig. 1A). The MLI (mean liner intercept) between the CS-exposed group and the control group showed no significant difference at the end of the 8^th^ week (Fig. 1B and C). However, after 16 weeks of CS-exposure, the MLI was significantly higher in the CS-exposed group than in the control group (Fig. 1B and C). More statistical details can be found in Supplementary Table S1.

#### CS-exposed rats with higher serum β_2_-AAb levels showed higher MLI values than the CS-exposed rats with lower serum β_2_-AAb levels

After 16 weeks of CS exposure, we divided the 12 CS-exposed rats into two subgroups, and the median value of all the serum β_2_-AAb levels was chosen as the cut-off value. The lower group included the 6 CS-exposed rats with relatively lower serum β_2_-AAb levels, and the higher group included the 6 CS-exposed rats with relatively higher serum β_2_-AAb levels. The higher group showed significantly higher serum β_2_-AAb levels (Fig. 1D) and higher MLI values (Fig. 1E) than the lower group. More statistical details can be found in Supplementary Table S1.

### Measurements for the active-immune and CS-exposed-active-immune rat model

#### Rats immunized with the ECL_II_ peptide showed higher β_2_-AAb levels in their sera and emphysema-like changes in their lungs

**Figure 2:**
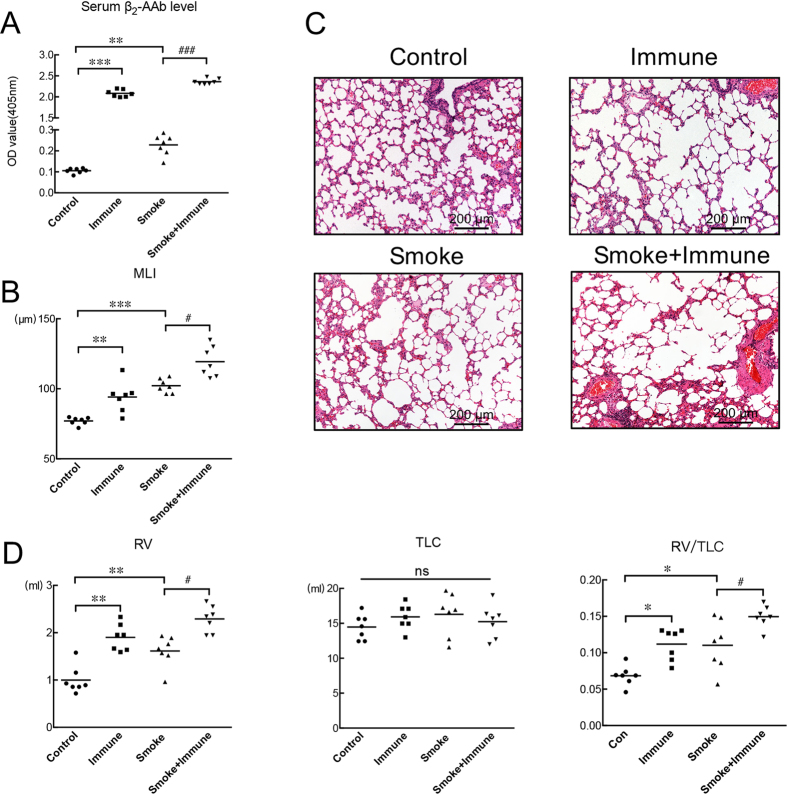
Measurements after control or passive-smoking rats were immunized or not with β_2_-AR ECL_II_ peptides for 16 weeks. (**A**) SE-ELISAs were performed to detect the levels of β_2_-AAbs in the serum samples of rats from the four groups: Control group (Control), Active-Immune group (Immune), Passive-smoking group (Smoking), and Passive-smoking-active-immune group (Smoking + Immune). Analyses were conducted between the Control group and Immune group/Smoking group as well as between the Smoking group and Smoking + Immune group. (**B**) Lung-section MLI measurements statistical results from the Control group and Immune group as well as from Smoking group and Smoking + Immune group. (**C**) Section of lung from a control rat showing normal alveolar structures and sections from rats in the other three groups showing enlarged airspaces. Haematoxylin-eosin staining, scale bar = 200 μm. (**D**) Statistical analysis of lung function parameters (RV (residual volume), TLC (total lung capacity) and RV/TLC (residual volume/total lung capacity) ratio) between the Control group and Immune group/Smoking group as well as between the Smoking group and Smoking + Immune group. *p < 0.05, **p < 0.01, ***p < 0.001; Control group vs Immune group/Smoking group (n = 7). ^###^*p* < 0.001 and ^#^*p* < 0.05; Smoke group vs Smoke + Immune group (n = 7), ns means no significant difference.

After the 16-week immunization with the β_2_-AR ECL_II_ peptide, significantly increased levels of β_2_-AAbs were present in the sera of the rats (Fig. 2A). The lungs of rats immunized with ECL_II_ peptides showed enlarged alveolar airspaces, and they had worse alveolar airspace destruction and increased MLI values when compared with the lungs of control rats (Fig. 2B and C). Pulmonary function test results showed that the residual volume (RV) and the RV/TLC (total lung capacity) ratio were significantly higher in the immunized group than in the control group (Fig. 2D), while TLC showed no statistical changes between the two groups (Fig. 2D). More statistical details can be found in Supplementary Tables S2 and S3.

#### CS-exposed rats immunized with the β_2_-AR ECL_II_ peptide exhibited higher serum β_2_-AAb levels and showed higher MLI values than the CS-exposed rats not immunized with the β_2_-AR ECL_II_ peptide

After CS exposure for 16 weeks, rat showed increased serum β_2_-AAb levels (Fig. 2A), MLI values (Fig. 2B and C) and RVs, RV/TLC values than that in control rats (Fig. 2D). And after CS exposure and immunization with the β_2_-AR ECL_II_ peptide for 16 weeks, rats showed higher serum β_2_-AAb levels than were found in rats only exposed to cigarette smoke (Fig. 2A). Similarly, the CS-exposed rats immunized with the β_2_-AR ECL_II_ peptide showed worse alveolar airspace destruction (Fig. 2C), higher MLI values (Fig. 2B), larger RVs and higher RV/TLC ratios (Fig. 2D) than rats only exposed to cigarette smoke. TLC showed no statistical changes among the groups (Fig. 2D). More statistical details can be found in Supplementary Tables S2 and S3.

### Studies on human subjects

#### Baseline characteristics and correlation analysis of all smokers

**Figure 3:**
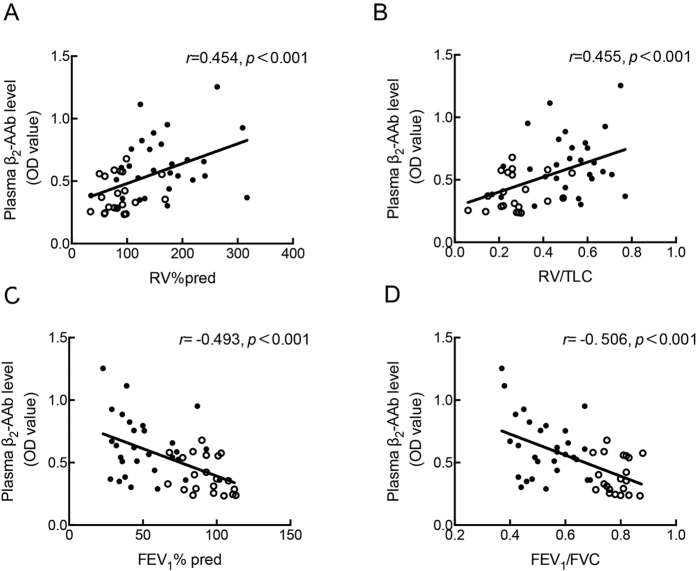
Correlation analysis between the plasma β_2_-AAb level and the lung function parameters. Plasma β_2_-AAb level were positively correlated with RV% pred (**A**) and RV/TLC ratio(**B**) and negatively correlated with FEV_1_% pred(**C**) and FEV_1_/FVC(**D**) ratio in 50 smokers. RV% pred (residual volume/residual volume predicted percentage), RV/TLC (residual volume/total lung capacity), FEV_1_% pred (percent of forced expiratory volume in 1 second), FEV_1_/FVC (forced vital capacity). Pearson or Spearman correlation analysis, n = 50. The hollow dots represent smokers without COPD, and the solid dots represent those with COPD.

The following parameters: FEV_1_% pred (percent of forced expiratory volume in 1 second), FEV_1_/FVC (forced expiratory volume in 1 second /forced vital capacity) ratio, RV% pred (residual volume/residual volume predicted percentage) and the RV/TLC (residual volume/total lung capacity) ratio were obtained from all smokers. To assess the correlation between plasma β_2_-AAb levels and the parameters obtained from the lung function tests, Pearson or Spearman correlation analyses were performed, and the corresponding scatter diagrams are shown in Fig. 3. The results show that plasma β_2_-AAb levels were significantly and positively correlated with RV% pred (*r* = 0.454, *p* < 0.001) and the RV/TLC ratio (*r* = 0.455, *p* < 0.001) and were negatively correlated with FEV_1_% pred (*r* = −0.493, *p* < 0.001) and the FEV_1_/FVC ratio (*r* = −0.506, *p* < 0.001).

The multivariable multivariate linear regression model adjusted for age, BMI (body mass index), and smoking history (expressed in pack years), demonstrated that plasma β_2_-AAb levels were independently correlated with RV% pred, RV/TLC ratio, FEV1% pred, FEV_1_/FVC ratio. More statistical details can be found in Supplementary Table S4.

#### Subgroup studies of low-β_2_-AAb and high-β_2_-AAb smokers

**Figure 4:**
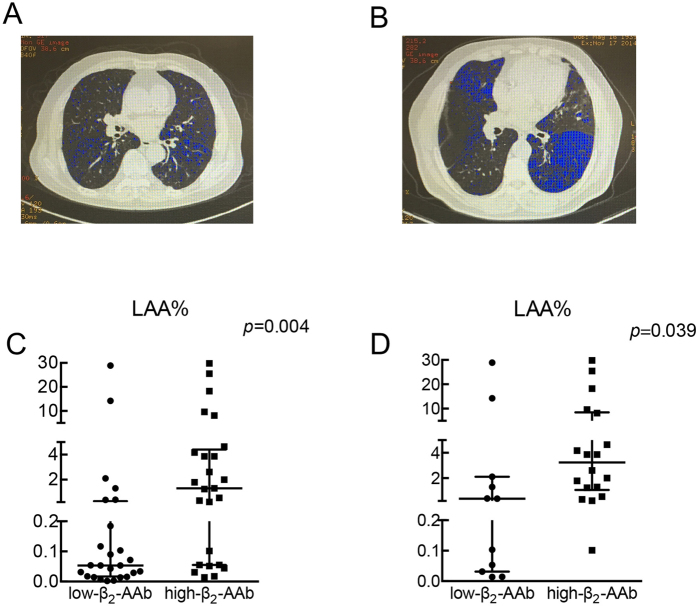
Smokers/COPD patients with relatively higher plasma β_2_-AAb levels exhibited worse LAA%. Fifty smokers were divided into low-β_2_-AAb and high-β_2_-AAb groups. (**A**) and (**B**) show one slice of chest CT scans from a low-β_2_-AAb smoker (**A**) and a high-β_2_-AAb smoker (**B**); the blue regions in the slices indicate the low attenuation area under −950 HU. (**C**) and (**D**) show the distributions (median with interquartile range are shown) and results of the statistical analysis of the LAA% comparing low-β_2_-AAb and high-β_2_-AAb smokers (**C**) and COPD patients (**D**). (n = 25 for low-β_2_-AAb and high-β_2_-AAb smokers, n = 11 for low-β_2_-AAb COPD patients and n = 18 for high-β_2_-AAb COPD patients). HU: Hounsfield units. LAA%: the percentage of low attenuation area under −950 HU in the chest CT scans.

The division smokers into low-β_2_-AAb and high-β_2_-AAb groups is described in the methods. Table 1 shows the baseline characteristics of the 2 groups. Subjects in the two groups were of comparable age, BMI, and smoking history (expressed in pack-years). The high-β_2_-AAb smokers showed worse RV% pred, RV/TLC ratios, FEV1% pred, FEV1/FVC ratios and DLCO% pred (diffusing capacity of the lungs for carbon monoxide/predicted values) than the low-β_2_-AAb smokers. All participants underwent HRCT (high resolution CT) scans. Additional tests compared the LAA% (low attenuation area percentage under −950 HU) values of the smokers in two subgroups. As shown in Fig. 4A–C, the high-β_2_-AAb smokers showed worse LAA% values than the low-β_2_-AAb smokers. More statistical details can be found in Supplementary Table S6.

#### Subgroup studies of low-β_2_-AAb and high-β_2_-AAb COPD patients

In the low-β_2_-AAb group, 11 of the 50 smokers were diagnosed with COPD, and in the high-β_2_-AAb group, 18 were diagnosed with COPD. As shown in Table 2, the age, pack-years, and BMI values of low-β_2_-AAb and high-β_2_-AAb COPD patients did not show significant differences. The high-β_2_-AAb COPD patients showed significantly worse RV% pred and RV/TLC values than the low-β_2_-AAb COPD patients. As shown in Fig. 4D, the 18 high-β_2_-AAb COPD patients showed worse LAA% values than the 11 low-β_2_-AAb COPD patients. While the FEV_1_% pred, FEV_1_/FVC ratio and DLCO% pred showed no significance between the two groups. More statistical details can be found in Supplementary Table S6.

## Discussion

In the present study, we firstly demonstrated the presence and increased expression of β_2_-AAbs against the second extracellular loop of the β_2_-AR in rats with CS-exposure-induced emphysema. We found that serum β_2_-AAb levels significantly increased before alveolar airspaces became enlarged in CS-exposed rats, and CS-exposed rats with higher serum β_2_-AAb levels had higher MLI values than CS-exposed rats with lower serum β_2_-AAb levels. In the active-immune rat model, we found significantly increased serum β_2_-AAb levels in immunized rats, and the immunized rats showed larger MLI and RV values and RV/TLC ratios than the control rats. The rats subjected to both CS-exposure and immune activation showed larger MLI values and RV/TLC ratios than the rats subjected only to CS-exposure. In our clinical studies, we observed that plasma β_2_-AAb levels showed a significant and positive correlation with RV% pred and RV/TLC ratios among smokers. Both smokers and COPD patients with higher plasma β_2_-AAb levels showed higher RV%, RV/TLC ratio and LAA% values than smokers or COPD patients with lower plasma β_2_-AAb levels. Based on the results in both humans and rats, we suggest that circulating β_2_-AAbs are associated with alveolar destruction and increases in RV.

Our study was primarily based on animal models. We previously reported the effectiveness of 16 weeks of CS-exposure in inducing emphysema and COPD-like changes based on the pathological and pulmonary function changes that occur in rat lungs^13^,^20^. In this study, we used this model to analyse whether β_2_-AAb levels are increased before the development of smoking-induced emphysema. In addition, studies on β_1_-adrenergic receptor (β_1_-AR) autoantibodies (β_1_-AAbs) have indicated that active immunization with synthetic peptides corresponding to the second extracellular loop of the β_1_-AR induces a marked production of β_1_-AAbs, which have similar biological and immunological properties as in the plasma of DCM (dilated cardiomyopathy) patients^21^. Therefore, a rat model of active immunization using a synthetic peptide corresponding to the ECL_II_ of the rat β_2_-AR was established to perform additional investigations *in vivo*. Using the active-immune rat model, our previous studies have shown that the injection of the adjuvant alone has no effect on lung structure or function. In this study, we thoroughly mixed the peptide with the adjuvant and repeatedly injected the mixture into rats to maintain β_2_-AAb production throughout the experimental period. We found that CS-exposed rats immunized with the β_2_-AR ECL_II_ peptide, who had higher serum β_2_-AAb levels, exhibited more extensive alveolar airspace destruction than rats subjected only to CS-exposure. CS-exposed rats with higher serum β_2_-AAb levels showed higher MLI values than CS-exposed rats with lower serum β_2_-AAb levels. These findings are in accordance with the findings of our clinical study showing that high-β_2_-AAb smokers and COPD patients were found to have larger LAA% values. These data indicate that β_2_-AAbs may be involved in the destruction of alveolar airspaces.

Previous studies have found that age, smoking history (in pack-years) and BMI are all related to autoantibody production and lung function in COPD and emphysema patients^7^. In our subgroup analysis, all of these parameters were matched between the groups, and all subjects were current male smokers, so the effects of smoking status and gender were also avoided. Moreover, age, BMI (body mass index), smoking history (expressed in pack years) were also adjusted in the multivariable multivariate linear regression model, which strengthened our study. In addition, lung function tests and CT scans were performed, which provided stronger evidence to support our findings. Smoking exposure is able to provoke oxidative stress and upregulate immune genes, thereby leading to autoimmune responses. Immune responses in smokers vary, and the identity and nature of the antigens that drive the production of autoantibodies in smokers remains unknown. In COPD, lymphoid follicles in the lungs may be the result of smoke exposure, and they are associated with disease severity. B cells are the predominant cell type in these follicles, and the proliferation of B cells within the germinal centres of these follicles shows antigen-specific characteristics^22^. Carbonyl-modified proteins may be the result of long-term smoke exposure, and studies have indicated that levels of antibodies against carbonyl-modified proteins correlate with the severity of COPD^23^. Our previous studies also indicated that long-term smoke exposure affected the expression of β_2_-ARs in the lung^20^. However, whether this is the source of self-antigens still needs to be determined.

Some autoantibodies found in association with emphysema or COPD have been shown to have little clinical or pathological importance. Previous observational studies have demonstrated higher titres of circulating antinuclear antibodies and anti-tissue antibodies in some COPD patients, and only anti-tissue antibodies were found to be related to impaired lung function, but the underlying mechanism has not been studied further^3^. A previous study also showed an increased expression of xenogeneic EC autoantibodies. Rats injected intraperitoneally with xenogeneic ECs were found to produce antibodies against ECs, and active immunization with ECs or the passive injection of anti-EC autoantibodies induced emphysema and cell apoptosis. In addition, MMP-9 and MMP-2 activation were found to take part in this process^24^. Currently, protease/anti-protease imbalances and cell apoptosis in the lungs are all proposed as major mechanisms resulting in emphysema, and MMP-9 has been recognized as one of the important elastases that contribute to the development of emphysema^25^. Common autoantibodies induce regular immune responses, leading to the destruction of the affected tissue, whereas autoantibodies target adrenergic receptors showing functional activity^15^. Numerous studies have shown that DCM patients may produce β_1_-AAbs, which bind to and activate the receptor, provoking biological responses related to the pathogenesis of DCM^26^,^27^,^28^,^29^,^30^. β_1_-AAbs may induce cell death *in vitro*, whereas the neutralization of β_1_-AAb aptamers has a protective effect against cell death. *In vivo*, the application of immunoadsorption to remove β_1_-AAbs from the blood of DCM patients has long-lasting benefits, including a higher left ventricular ejection fraction^28^, less oxidative stress^29^, and a better 5-year survival rate^30^. Only a few studies have focused on β_2_-AAbs. These studies have indicated that one type of human monoclonal β_2_-AAb against the N terminus of the ECL_II_ peptide displays agonist-like functions *in vitro*^31^. The activation of β_2_-ARs is likely to have beneficial effects for relieving airflow limitations, although the activation of β_2_-ARs in the absence of pro-inflammatory stimuli has been shown to produce increased IL-1β and IL-6 transcripts in macrophage cell lines^10^. Macrophages play important roles in the development of emphysema, and IL-6 and IL-1β are both involved in emphysema^32^. Salbutamol, a β_2_-AR agonist, has been shown to promote MMP-9 expression in the bronchoalveolar lavage fluid of ARDS patients^33^. However, classical β-AR agonists and the β-AR autoantibodies have different binding sites (hydrophobic pockets and extracellular loops, respectively)^15^, and the β_2_-AAbs identified in our study were polyclonal antibodies. Determining the exact functions of β_2_-AAbs *in vivo* needs further investigation.

DLCO% pred, which assesses the potential of the lung for gas exchange, is decreased when alveolar airspaces are damaged in smokers^34^,^35^. We found that the plasma β_2_-AAb levels in smokers were correlated with DLCO% pred and not with β_1_-AAbs (see Supplementary Table S4 and S6), and smokers wither higher plasma β_2_-AAb levels showed worse DLCO% pred, which may indicate more extensive alveolar airspace damage. We also noticed than in our correlation studies, plasma β_2_-AAbs in smokers were negatively correlated with FEV_1_ and the FEV_1_/FVC ratio. However, in additional studies, we found that that plasma β_1_-AAbs in smokers were also negatively correlated with FEV_1_% pred, but were not significantly correlated with RV% pred and the RV/TLC ratio (see Supplementary Fig. S3). In addition, we found that the β_1_-AAbs were not significantly greater in passive-smoking rats than in control rats after CS-exposure for 16 weeks (see Supplementary Fig. S1). Considering the similarity of our results with those of a study focusing on the relationships between autoantibodies and lung function^36^, the autoantibodies associated with emphysema may be antigen specific. We noticed that the serum β_2_-AAb levels in active-immune rats were much higher than those in passive-smoking rats, while the morphological analysis and lung function tests did not indicate worse alveolar airspace destruction in the active-immune groups than in the passive-smoking rats. However, we did notice that passive smoking rats immunized with ELC_II_ peptide exhibited worse RV% pred, RV/TLC and MLI values than rats subjected to passive-smoking alone. Passive-smoking and active-immune stimulation may involve different pathophysiological processes.

TLC is composed of several parts, such as residue volume and the IC (inspiratory capacity) values. According to a previous study^34^, the destruction of alveolar airspace may or may not be associated with airflow limitation, and RV is generally the first to increase, followed by other parameters, such as TLC^37^. In our clinical studies, we did notice that smokers with higher plasma β_2_-AAb levels exhibited worse FEV_1_/FVC and FEV_1_% pred. Because of limited applications in rat lung function tests, the relationship between β_2_-AAbs and airflow limitation needs further study. At the same time, we notice that the IC/TLC value may reflect the lung hyperinflation^38^. And our results in animals are in accordance with the study that the CS-exposed rats immunized with β_2_-AR ECL_II_ peptide showed decreased IC/TLC values than CS-exposed rats (see Supplementary Fig. S2).

There are limitations in this study that should be noted. 1) The homology of the peptide sequence of the ECL_II_ of the β_1_-AR between humans and rats was 100%, whereas the homology of the β_2_-AR ECL_II_ sequence was 98%. 2) It is not yet clear how to effectively influence the production of β_2_-AAbs, and although neutralized peptides proved to be useful *in vitro*, their use would induce more β_2_-AAb production *in vivo*. 3) Considering that the majority of COPD and emphysema patients in our country are male smokers, our clinical study was based on a relatively small sample of male smokers, and whether the effects of β_2_-AAbs are related to gender needs further study. 4) In addition, whether β_2_-AAb expression increases and is involved in COPD-related diseases in non-smokers or in genetics-based emphysema, such as that caused by an alpha-1 antitrypsin deficiency, are not known.

Our study extends the knowledge of the relationships of autoantibodies with emphysema and COPD, which will benefit future studies. We suggest that higher circulating β_2_-AAb levels may be associated with worse alveolar airspace destruction, and may aggravate smoking-related lung injuries. Our research suggests that β_2_-AAbs may act as a biomarker for increased alveolar airspace destruction in male smokers, which may help in identifying new therapies for treating emphysema.

## Methods

### Animal model

Protocols were approved by the Ethics Committee for Animal Experiments of Peking University (permit No: LA2010-004), and the methods were strictly carried out in accordance with the approved guidelines. Rats were obtained from the animal centre of Peking University Health Science Centre and were housed as previously reported^13^. Thirty-six male Sprague-Dawley rats (SD rats, 7 weeks old) were used in our preliminary test of the passive-smoking model: (1) eighteen Control rats were housed in clean air and were not subjected to CS-exposure; and (2) eighteen CS-exposed rats were exposed to the smoke from 30 cigarettes (Du Bao brand, China) twice per day, 6–7 times per week, from the 1^st^ to the 8^th^ or 16^th^ week using the BUXCO animal CS-exposure system (DSI, USA). Six control rats and six passive-smoking rats were sacrificed at the end of the 8^th^ week and others were sacrificed at the end of the 16^th^ week. In the passive-smoking-active-immune model, twenty-eight male SD rats (7 weeks old) were divided into 4 groups: (1) seven Control rats were housed in clean air and were not subjected to CS-exposure or peptide injections; (2) seven CS-exposed rats were exposed to smoke as described above; (3) seven rats in the active-immune group were housed with clean air and were given hypodermic injections of rat β_2_-AR ECL_II_ peptides (1 μg/100 g, synthesized by a contractor: Shanghai GL Biochem. Ltd. China) combined with a complete adjuvant (Sigma, USA) for the first injection and with an incomplete adjuvant (Sigma, USA) for subsequent injections, which were repeated twice a week from the 1^st^ to the 16^th^ week; and (4) seven rats were exposed to both cigarette smoke and administered β_2_-AR ECL_II_ peptide immunizations from the 1^st^ to the 16^th^ week. Measurements of the mean linear intercept (MLI) in rats were performed as previously reported^24^.

### Lung function in rats

The equipment was calibrated before use^39^. Rats were anaesthetized with 1% pentobarbital sodium (0.4 mg/100 g, intra-peritoneal injection; Sigma, USA) prior to surgery. Tracheostomies were performed with a standard catheter provided with the Buxco equipment (DSI, USA). Rats were placed in a body plethysmograph and connected to a computer-controlled ventilator after tracheostomy.

### Peptide synthesis

The peptides corresponding to the sequence (amino acid residues 172–198) of the ECL_II_ peptide of the human β_2_-AR (H-W-Y-R-A-T-H-Q-E-A-I-N-C-Y-A-N-E-T-C-C-D-F-F-T-N-Q-A), rat β_2_-AR (H-W-Y-R-A-T-H-K-Q-A-I-D-C-Y-A-K-E-T-C-C-D-F-F-T-N-Q-A) and β_1_-AR (H-W-W-R-A-E-S-D-E-A-R-R- C-Y-N-D-P-K-C-C-D-F-V-T-N-R-C) were synthesized using an automated peptide synthesizer via solid-phase methods. Peptide purity was assessed via high-performance liquid chromatography (HPLC) using an automated amino-acid analyser. Peptide preparations were found to be 98% pure. The process was performed by a contractor: Shanghai GL Biochem, China.

### Rats tissue treatment and the collection of serum samples

Blood samples were taken from rats via the vessels in their tails every four weeks during the experiment or from the abdominal aorta before rats were sacrificed and were centrifuged at 3000 rpm for 15 minutes at 4 °C without an anticoagulant to obtain serum samples. Serum samples were stored at −80 °C until further study. At each time point, the serum β_2_-AAb levels of rats were assessed via SE-ELISA. The left lobe of the lung of each rat was clamped at the hilum and preserved in 4% paraformaldehyde for histological analysis. Lung tissues were embedded in paraffin and stained with haematoxylin and eosin for pathological analyses.

### SE-ELISA

Fifty microliters of ECL_II_ peptide (5 μg/ml) in a 0.1 M Na_2_CO_3_ solution (pH 11.0) was coated onto a 96-well microplate overnight at 4 °C. The wells were then saturated with PBS supplemented with 5% bovine serum and 0.1% Tween 20 (PMT). Human plasma samples were diluted 80 times, and rat serum samples were diluted 10,000 times before use. Fifty microliters of diluted/undiluted samples in PMT were allowed to react with the peptide for 1 hour at 37 °C. After washing three times with PBS (including 0.05% Tween 20, washing buffer), 0.05 ml of biotinylated rabbit anti-human/rat IgG antibody (1:1000 dilution in PMT) was added and allowed to react for 1 hour at 37 °C. After three washes, the bound biotinylated antibody was detected following the incubation of the plates with a streptavidin-peroxidase (1 μg/ml) solution in PMT for 1 hour. This was followed by three washes and the addition of the substrate (2.5 mmol/L H_2_O_2_, 2 mmol/L ABTS, Sigma, USA). Optical densities (ODs) were read at 405 nm after 30 minutes in a standard microplate spectrophotometer and were used to indicate the level of β_2_-AAbs according to the researches in β_1_-AAbs^40^ and other autoantibodies^24^,^41^. All samples were measured in duplicate wells, and the average values were used. One sample was selected for inclusion on each ELISA plate to control for interassay variability.

### Study population and emphysema assessment

Peking University Third Hospital Research and Development Department approved the study protocol (NO. IRB00001052-08089), and the methods were strictly carried out in accordance with the approved guidelines. Sixty-five male current smokers were recruited between October and December 2014. Individuals who had an α1-anti-trypsin deficiency, a history of physician-diagnosed asthma and other autoimmune diseases (rheumatoid arthritis, systemic lupus erythematosus, Crohn’s disease), Chagas’ cardiomyopathy, open-angle glaucoma or regional pain syndrome were excluded from the study. COPD patients were also excluded if they had experienced an exacerbation of the disease within the previous 6 weeks. Individuals were also excluded if they refused to perform chest CT scans. At last, fifty individuals were recruited for further studies. Of the 50 male current smokers in this study, 29 were classified as having COPD, and 21 were defined as smokers without airway flow restriction. Written informed consent was obtained from all participants. Data on age, height, weight, and smoking history were collected by asking the subjects. FEV_1_% pred, RV% pred, and the FEV_1_/FVC and RV/TLC ratios were measured via pulmonary function tests (Sensor Medics, Yorba Linda, CA, USA). All participants underwent chest CT scans, and the percentage of the low attenuation area below −950 Hounsfield units (HU)^42^ (LAA%) was measured using AW version 4.5 software (GE healthcare, Fairfield, CT, USA) to determine the extent of emphysema. Diagnoses of COPD followed the guidelines of the Global Initiative for COPD (GOLD), which were revised in 2016. Blood samples (approximately 4 ml) were collected from all participants via an antecubital vein into a vacuum tube containing ethylenediaminetetraacetic acid (EDTA) and were centrifuged at room temperature within half an hour from the time of collection. The separated plasma samples were aliquoted into sterile microcentrifuge tubes (500 μl in each vial) and then stored at −80 °C. In addition, we used SE-ELISAs to determine the plasma β_2_-AAb levels in each sample after a dilution of 1:80. The median of all plasma β_2_-AAb values (OD values at 405 nm) were used as the cut-off value to divided the smokers into two sub-groups: the low-β_2_-AAb group and the high-β_2_-AAb group. Flow diagram of the study can be found in Supplementary Figure S4.

### Data analysis

Results are expressed as the mean ± SD or mean values with individual data points and were analysed for statistical significance using GraphPad Prism (GraphPad) and SPSS 11.5. The results with a *p* < 0.05 were considered to be statistically significant. Shapiro-Wilk tests were applied to assess the normality of the distribution of continuous variables in each group. In addition, Levene’s tests were applied to assess the homogeneity of variance. If the *p* values for both tests were greater than 0.05, T-tests or ANOVAs were conducted. Direct comparisons between two groups were performed using non-parametric Mann-Whitney tests (between two groups) or Kruskal-Wallis H tests (more than two groups) when the data sets were not normally distributed. In clinical studies, correlation analyses were performed using the Pearson or Spearman method; multivariate linear regression model were also employed to adjust the effect of confounders.

## Additional Information

**How to cite this article:** Hu, J.-y *et al*. Increased circulating β_2_-adrenergic receptor autoantibodies are associated with smoking-related emphysema. *Sci. Rep.* **7**, 43962; doi: 10.1038/srep43962 (2017).

**Publisher's note:** Springer Nature remains neutral with regard to jurisdictional claims in published maps and institutional affiliations.

## Supplementary Material

Supplementary Information

## Figures and Tables

**Table 1:** Comparison of demographic characteristic and clinical variables between low-β_2_-AAb and high-β_2_-AAb smokers.

Variable	Low-β_2_-AAb group	High-β_2_-AAb group	*p* value
	n = 25	n = 25	
Age (years)	56 ± 13	62 ± 11	0.055
FEV_1_% pred	80 ± 26	58 ± 25	0.005
FEV_1_/FVC	0.68 ± 0.13	0.59 ± 0.15	0.028
RV% pred	104 ± 58	156 ± 66	0.002
RV/TLC	0.34 ± 0.17	0.49 ± 0.16	0.002
DLCO% pred	78.16 ± 20.14	62.48 ± 21.46	0.010
BMI (kg/m^2^)	23.65 ± 3.59	23.80 ± 3.41	0.898
Pack years	29 ± 18	36 ± 26	0.478
Plasma β_2_-AAb level	0.35 ± 0.10	0.70 ± 0.18	＜0.001

Data are presented as the mean ± SD. Abbreviations: β_2_-AAb = β_2_-adrenergic receptor autoantibody; BMI, body mass index; FEV_1_, percent of forced expiratory volume in 1 second; FEV_1_/FVC, forced expiratory volume in 1 second /forced vital capacity; RV% pred, residual volume/residual volume predicted percentage; RV/TLC, residual volume/total lung capacity. DLCO% pred, diffusing capacity of the lungs for carbon monoxide/predicted values. Plasma β_2_-AAb levels are presented as the OD values in 405 nm.

**Table 2:** Comparison of demographic characteristic and clinical variables between low-β_2_-AAb and high-β_2_-AAb COPD patients.

Variable	Low-β_2_-AAb group	High-β_2_-AAb group	*p* value
	n = 11	n = 18	
Age (years)	61 ± 13	65 ± 10	0.448
FEV_1_% pred	59 ± 25	47 ± 18	0.121
FEV_1_/FVC	0.56 ± 0.10	0.51 ± 0.09	0.197
RV% pred	129 ± 75	181 ± 56	0.013
RV/TLC	0.43 ± 0.18	0.56 ± 0.12	0.030
DLCO% pred	67.18 ± 21.87	57.67 ± 20.89	0.252
BMI (kg/m^2^)	22.47 ± 3.05	23.60 ± 3.28	0.363
Pack years	38 ± 21	41 ± 28	0.787
Plasma β_2_-AAb level	0.41 ± 0.10	0.75 ± 0.19	＜0.001

Data are presented as the mean ± SD. Abbreviations: β_2_-AAb = β_2_-adrenergic receptor autoantibody; COPD, chronic obstructive pulmonary disease; BMI, body mass index; FEV_1_% pred, percent of forced expiratory volume in 1 second; FEV_1_/FVC, forced expiratory volume in 1 second /forced vital capacity; RV% pred, residual volume/residual volume predicted percentage; RV/TLC, residual volume/total lung capacity, DLCO% pred, diffusing capacity of the lungs for carbon monoxide/predicted values. Plasma β_2_-AAb levels are presented as the OD values in 405 nm.
