# Author Correction: Increased circulating β2-adrenergic receptor autoantibodies are associated with smoking-related emphysema

**DOI:** 10.1038/s41598-020-63071-y

**Published:** 2020-04-10

**Authors:** Jia-yi Hu, Bei-bei Liu, Yi-Peng Du, Yuan Zhang, Yi-wei Zhang, You-yi Zhang, Ming Xu, Bei He

**Affiliations:** 1 Department of Respiratory Medicine, Peking University Third Hospital, Beijing, China; 2 Department of Cardiology, Institute of Vascular Medicine, Peking University Third Hospital, Key Laboratory of Molecular Cardiovascular Sciences, Ministry of Education, Key Laboratory of Cardiovascular Molecular Biology and Regulatory Peptides, Ministry of Health; Beijing Key Laboratory of cardiovascular Receptors Research, Beijing, China

Correction to: *Scientific Reports* 10.1038/srep43962, published online 06 March 2017

This Article contains an error in Figure 2C, where the incorrect image was used for the 16-week control rat. The correct Figure 2 appears below as Figure 1.

**Figure 1:**
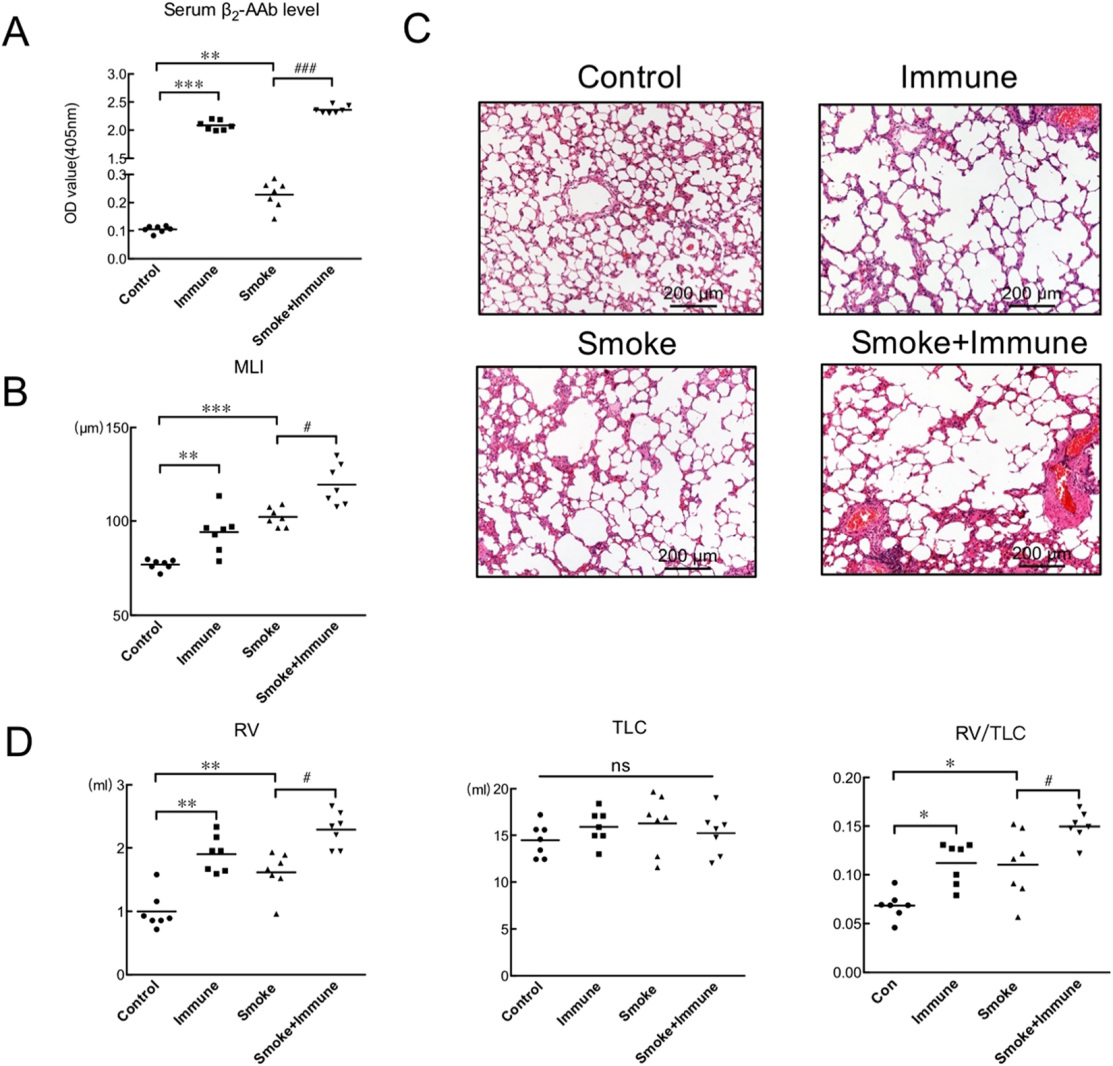
.

