# Supplementary material for: Increased circulating β2-adrenergic receptor autoantibodies are associated with smoking-related emphysema
Source: Sci Rep. 2017 Mar 6;7:43962. doi: 10.1038/srep43962 (PMC5338268; doi:10.1038/srep43962)
Supplement: Supplementary Information [file srep43962-s1.pdf]

Supplementary information:

Title: Increased circulating β_2-adrenergic receptor autoantibodies are associated with smoking-related emphysema

Authors: Jia-yi Hu[1]; Bei-bei Liu[1]; Yi-peng Du[1]; Yuan Zhang[1]; Yi-wei Zhang[1]; You-yi Zhang[2]; Ming Xu [2*]; Bei He[1*]

Affiliations:

1. Department of Respiratory Medicine, Peking University Third Hospital, Beijing, China.

2. Department of Cardiology, Institute of Vascular Medicine, Peking University Third Hospital, Key Laboratory of Molecular Cardiovascular Sciences, Ministry of Education, Key Laboratory of Cardiovascular Molecular Biology and Regulatory Peptides, Ministry of Health and Beijing Key Laboratory of cardiovascular Receptors Research, Beijing, China.

***Corresponding author: Bei He:** puh3_hb@bjmu.edu.cn **Ming Xu:** xuminghi@bjmu.edu.cn

Telephone: Bei He: 86-15611908102 **Ming Xu:** 86-10-82265519

Address: No. 49, North Huayuan Road, Haidian District, 100191, Beijing, China.

Email address for all: Jia-yi Hu (judy8hu@126.com); Bei-bei Liu (liubeibei416@126.com); Yi-peng Du (billydu@sina.com); Yuan Zhang (yuanzhang1129@163.com); Yi-wei Zhang (1511110305@pku.edu.cn); You-yi Zhang (zhangyy@bjmu.edu.cn).

Figure S1:

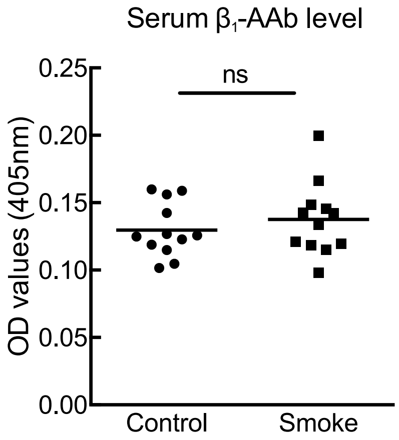

Figure S1. Serum β_1-AAb levels in rats were not significantly increased after 16 weeks of Cigarette smoke exposure. After 16 weeks of CS exposure, β_1-AAb serum values were not significantly increased in passive-smoking rats relative to levels in control rats. (n=12). More statistical details are present in Table S7. And ns means no significant difference.

Figure S2:

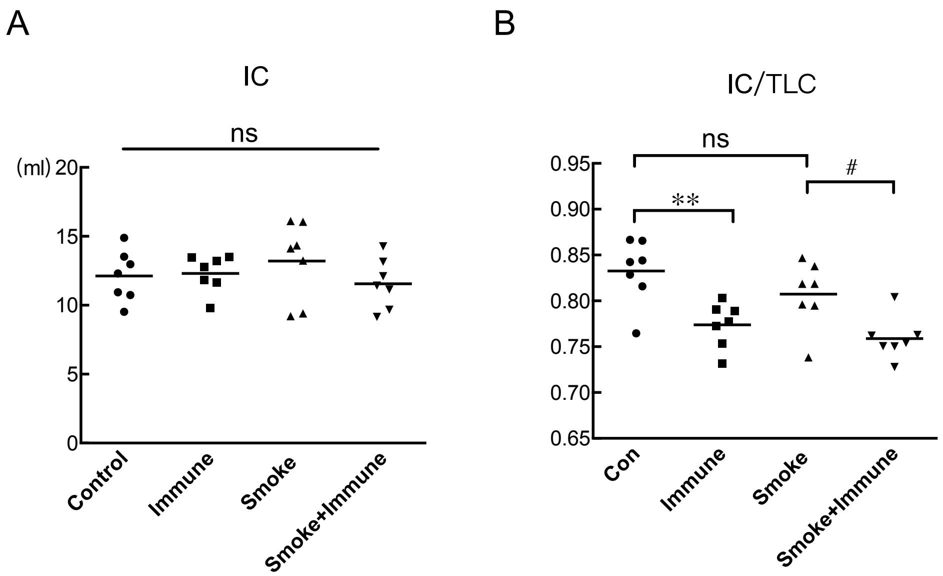

Figure S2. The IC values showed no significant difference among the rats in 4 groups (A). While rats showed decreased IC/TLC ratios after immunized with β_2-AR ECL_{II} peptides for 16 weeks than that in control rats (B). And CS-exposed rats immunized with β_2-AR ECL_{II} peptides also showed significantly decreased IC/TLC ratios than that in the CS-exposed rats. While ns means no significant difference. $^{**}p<0.01$, Control group vs Immune group (n=7). $^{\#}p<0.05$; Smoke group vs Smoke + Immune group (n=7).

Figure S3.

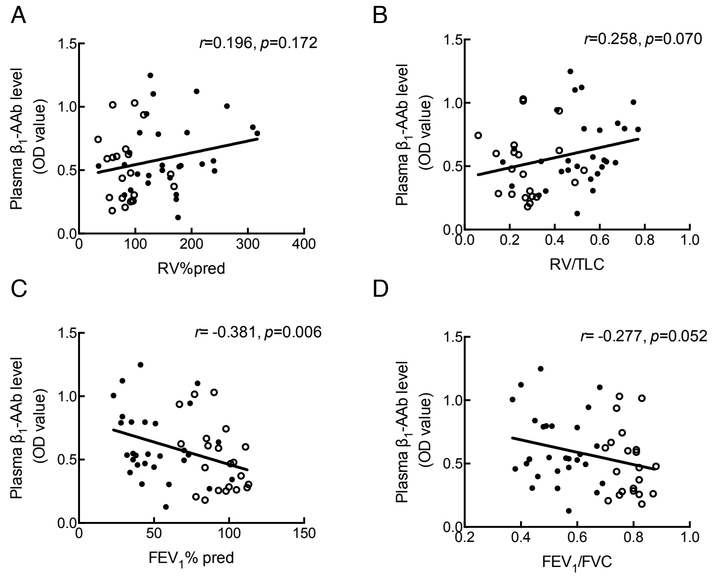

Figure S3. Correlation analysis between the plasma β_1-AAb level and the RV%pred, RV/TLC ratio, FEV_1%pred and FEV_1/FVC ratio in 50 smokers. Plasma β_1-AAb levels were negatively correlated with FEV_1%pred. RV%pred (residue volume/residue volume predicted percentage), RV/TLC (residue volume/total lung capacity), FEV_1%pred (percent of forced expiratory volume in 1 second), FEV_1/FVC (forced vital capacity). (Pearson or Spearman correlation method, n=50). The hollow dots represent smokers without COPD, and the solid dots represent those with COPD.

Figure S4.

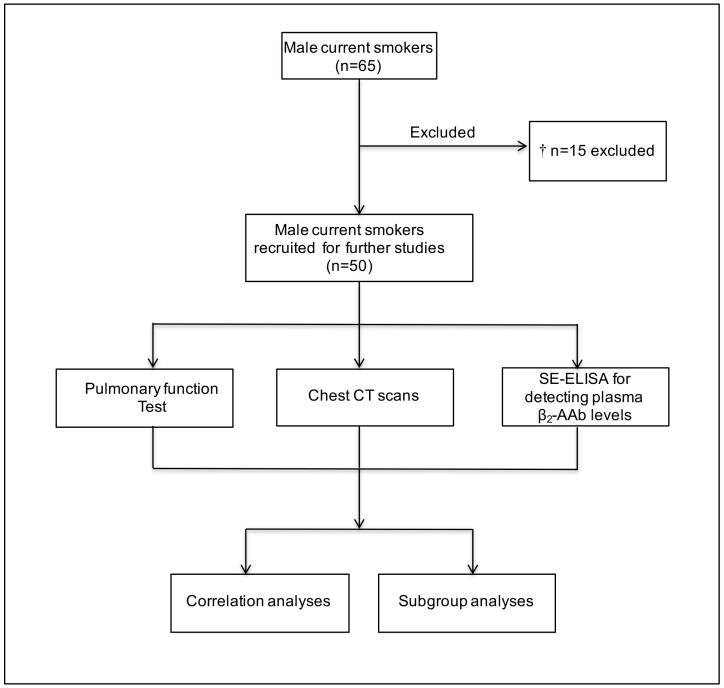

Figure S4. Flow diagram of the clinical study. Abbreviations: β_2-AAbs, β_2-adrenergic receptor autoantibody. †n=15 were excluded for the reasons listed below: a history of physician-diagnosed asthma and other autoimmune diseases. COPD patients were also excluded if they had experienced an exacerbation of the disease within the previous 6 weeks. Individuals were also excluded if they refused to perform chest CT scans.

Table S1: The distribution, homogeneity of variance test and comparison method between groups of continuous variables in the Figure 1.

Variable		Control group vs Smoke group				
		Shapiro-Wilk Test			Levene test	Comparison Method
		n value	Control	Smoking		
Serum β_2-AAb level	4th week	18	*p*=0.129	*p*=0.369	*p*=0.047	*p*=0.645, Adjusted T-Test
	8th week	18	*p*=0.062	*p*=0.233	*p*<0.001	*p*=0.012, Adjusted T-Test
	12th week	12	*p*=0.145	*p*=0.111	*p*=0.016	*p*=0.003, Adjusted T-Test
	16th week	12	*p*=0.239	*p*=0.080	*p*=0.110	*p*<0.001, T-Test
MLI values	8th week	6	*p*=0.437	*p*=0.160	*p*=0.223	*p*=0.223, T-Test
	16th week	12	*p*=0.924	*p*=0.478	*p*=0.283	*p*<0.001, T-Test

Variable		Lower group vs Higher group				
		Shapiro-Wilk Test			Levene test	Comparison Method
		n value	Lower	Higher		
Serum β_2-AAbs level	16th week	6	*p*=0.658	*p*=0.210	*p*=0.083	*p*=0.008, T-Test
MLI values	16th week	6	*p*=0.737	*p*=0.339	*p*=0.277	*p*=0.031, T-Test

Abbreviations: β_2-AAb, β_2-adrenergic receptor autoantibody; MLI, mean liner intercept.

Table S2: The distribution, homogeneity of variance test and comparison method between 4 groups of continuous variables in the Figure 2 and Figure S2.

Variable	Shapiro-Wilk Test				Levene test	Comparison Method
	Control	Immune	Smoke	Smoke+Immune		
Serum β_2-AAb level	p=0.394	p=0.318	p=0.685	p=0.148	p=0.005	p<0.001, Kruskal-Wallis H
MLI values	p=0.314	p=0.583	p=0.514	p=0.386	p=0.022	p<0.001, Kruskal-Wallis H
RV	p=0.061	p=0.318	p=0.669	p=0.537	p=0.630	p<0.001, ANOVA
TLC	p=0.510	p=0.865	p=0.305	p=0.776	p=0.779	†p=0.492, ANOVA
RV/TLC	p=0.549	p=0.068	p=0.732	p=0.725	p=0.107	p<0.001, ANOVA
IC	p=0.959	p=0.167	p=0.131	p=0.912	p=0.252	†p=0.513, ANOVA
IC/TLC	p=0.241	p=0.694	p=0.343	p=0.203	p=0.631	p=0.001, ANOVA

(†p=0.492 and †p=0.513, Multiple comparison were not proceeded)

Abbreviations: β_2-AAb, β_2-adrenergic receptor autoantibody; MLI, mean liner intercept; RV, residue volume; TLC, total lung capacity; IC: inspiratory capacity; RV/TLC, residue volume/total lung capacity ratio; IC/TLC: inspiratory capacity/ total lung capacity ratio.

Table S3: The multiple comparison method between the 4 groups listed in Figure 2 and Figure S2.

Variable	Control vs Immune	Control vs Smoke	Control vs Smoke+Immune	Immune vs Smoke	Immune vs Smoke+Immune	Smoke vs Smoke+Immune	Comparison Method
Serum β_2-AAb level	p<0.001	p=0.002	p<0.001	p<0.001	p<0.001	p<0.001	Dunnett's T3
MLI values	p=0.027	p<0.001	p<0.001	p=0.438	p=0.005	p=0.026	Dunnett's T3
RV	p=0.001	p=0.001	p<0.001	p=1.000	p=0.035	p=0.016	Bonferroni
RV/TLC	p=0.010	p=0.013	p<0.001	p=1.000	p=0.031	p=0.022	Bonferroni
IC/TLC	p=0.008	p=0.796	p=0.001	p=0.296	p=1.000	p=0.037	Bonferroni

Abbreviations: β_2-AAb, β_2-adrenergic receptor autoantibody; MLI, mean liner intercept; RV, residue volume; TLC, total lung capacity; IC: inspiratory capacity; RV/TLC, residue volume/total lung capacity ratio; IC/TLC: inspiratory capacity/ total lung capacity ratio.

Table S4: Relationship between plasma β_2-AAb level and clinical data assessed by univariate correlation analysis and multivariate linear regression model.

Variable	Univariate correlation analysis		Multivariate Linear Regression Model (Y= plasma β_2-AAb levels)									
			RV%pred		RV/TLC		FEV_1%pred		FEV_1/FVC		DLCO%pred	
	Pearson correlation	Spearman correlation	Beta	*p* value	Beta	*p* value	Beta	*p* value	Beta	*p* value	Beta	*p* value
RV%pred		r=0.454, p<0.001	0.002	0.001								
RV/TLC	r=0.455, p<0.001				0.673	0.003						
FEV_1%pred		r=-0.493, p<0.001					-0.005	0.001				
FEV_1/FVC		r=-0.506, p<0.001							-0.887	<0.001		
DLCO%pred	r=-0.389, p=0.005										-0.005	0.012
Age	r=0.303, p=0.033		0.005	0.042	0.003	0.287	0.002	0.539	0.001	0.664	0.004	0.219
BMI	r=0.002, p=0.989		-0.005	0.598	-0.003	0.723	0.001	0.884	0.008	0.395	0.006	0.512
SMOKE		r=0.076, p=0.601	-0.003	0.065	-0.002	0.159	-0.002	0.335	-0.001	0.523	-0.058	0.082

Abbreviations: β_2-AAb, β_2-adrenergic receptor autoantibody; RV% pred (residue volume/residue volume predicted percentage); RV/TLC (residue volume/total lung capacity ratio); FEV_1%pred (percent of forced expiratory volume in 1 second); FEV_1/FVC (forced expiratory volume in 1 second /forced vital capacity ration); DLCO%pred, diffusing capacity of the lungs for carbon monoxide/predicted values. BMI, body mass index; SMOKE, smoking history, in pack yeas.

Table S5. Comparison of demographic characteristic and clinical variables between low-β_2-AAb and high-β_2-AAb COPD patients after BD were applied.

Variable	Low-β_2-AAb group n=11	High-β_2-AAb group n=18	p value
FEV_1%pred	64.82 ± 26.09	50.50 ± 17.71	0.089
FEV_1/FVC	0.58 ± 0.10	0.53 ± 0.09	0.150

Data are presented as the mean ± SD. Abbreviations: β_2-AAb:β_2-adrenergic receptor autoantibody; COPD, chronic obstructive pulmonary disease; BMI, body mass index; FEV_1%pred (percent of forced expiratory volume in 1 second); BD: bronchodilators, in our study, salbutamol (200 μg) was applied for COPD diagnoses when the smokers got an FEV_1/FVC ration below 70%. Ventilation function were tested after the BD were administered.

Table S6: The distribution, homogeneity of variance test and comparison method between groups of variables in Table 1,2,S5 and Figure 4.

Variable	Smokers				COPD patients			
	Shapiro-Wilk Test		Levene test	Comparison Method	Shapiro-Wilk Test		Levene test	Comparison Method
	low	high			low	high		
Age	*p*=0.040	*p*=0.580	*p*=0.227	*p*=0.055, Mann-Whitney U	*p*=0.139	*p*=0.058	*p*=0.450	*p*=0.448, T-Test
FEV_1%pred	*p*=0.053	*p*=0.066	*p*=0.972	*p*=0.005, T-Test	*p*=0.525	*p*=0.104	*p*=0.175	*p*=0.121, T-Test
FEV_1/FVC	*p*=0.024	*p*=0.249	*p*=0.501	*p*=0.028, Mann-Whitney U	*p*=0.175	*p*=0.645	*p*=0.737	*p*=0.197, T-Test
RV%pred	*p*=0.000	*p*=0.803	*p*=0.248	*p*=0.002, Mann-Whitney U	*p*=0.046	*p*=0.560	*p*=0.679	*p*=0.013, Mann-Whitney
RV/TLC	*p*=0.295	*p*=0.197	*p*=0.981	*p*=0.002, T-Test	*p*=0.635	*p*=0.779	*p*=0.168	*p*=0.030, T-Test
DLCO%pred	*p*=0.174	*p*=0.429	*p*=0.418	*p*=0.01, T-Test	*p*=0.750	*p*=0.213	*p*=0.927	*p*=0.252, T-Test
FEV_1%pred(BD)					*p*=0.395	*p*=0.058	*p*=0.064	*p*=0.089, T-Test
FEV_1/FVC(BD)					*p*=0.575	*p*=0.415	*p*=0.474	*p*=0.150, T-Test
BMI	*p*=0.514	*p*=0.746	*p*=0.946	*p*=0.898, T-Test	*p*=0.680	*p*=0.054	*p*=0.480	*p*=0.363, T-Test
Pack years	*p*=0.098	*p*=0.001	*p*=0.324	*p*=0.478, Mann-Whitney U	*p*=0.544	*p*=0.012	*p*=0.354	*p*=0.787, Mann-Whitney
Plasma β_2-AAb level	*p*=0.036	*p*=0.001	*p*=0.003	*p*=0.000, Mann-Whitney U	*p*=0.211	*p*=0.104	*p*=0.047	*p*=0.000, Adjust T-Test
LAA%	*p*=0.000	*p*=0.000	*p*=0.164	*p*=0.004, Mann-Whitney U	*p*=0.000	*p*=0.000	*p*=0.921	*p*=0.039, Mann-Whitney

Abbreviations: β_2-AAb, β_2-adrenergic receptor autoantibody; RV%pred (residue volume/residue volume predicted percentage); RV/TLC (residue volume/total lung capacity ratio); FEV_1%pred (percent of forced expiratory volume in 1 second); FEV_1/FVC (forced expiratory volume in 1 second /forced vital capacity ration); LAA%, low attenuation area percentage; DLCO%pred (diffusing capacity of the lungs for carbon monoxide/predicted values);low, the low-β_2-AAb group; high, the high-β_2-AAb group; BD: bronchodilators, in our study, salbutamol (200 μg) was applied for COPD diagnoses when the smokers got an FEV_1/FVC ration below 70%. Ventilation function were tested after the BD were administered.

Table S7: The distribution, homogeneity of variance test and comparison method between groups in Figure S1.

Variable		Control group vs Smoke group				
		Shapiro-Wilk Test			Levene test	Comparison Method
		n value	Control	Smoke		
Serum β_1-AAb level	16th week	12	*p*=0.203	*p*=0.391	*p*=0.548	*p*=0.433, T-Test

Abbreviations: β_1-AAb, β_1-adrenergic receptor autoantibody.

Table S8: Relationship between plasma β_1-AAb level and clinical data assessed by univariate correlation analysis and multivariate linear regression model.

Variable	Univariate correlation analysis		Multivariate Linear Regression Model (Y= plasma β_1-AAb levels)									
			RV%pred		RV/TLC		FEV_1%pred		FEV_1/FVC		DLCO%pred	
	Pearson correlation	Spearman correlation	Beta	*p* value	Beta	*p* value	Beta	*p* value	Beta	*p* value	Beta	*p* value
RV%pred		*r*=0.196, *p*=0.172	0.001	0.110								
RV/TLC	*r*=0.258, *p*=0.070				0.454	0.106						
FEV_1%pred		*r*=-0.381, *p*=0.006					-0.004	0.015				
FEV_1/FVC		*r*=-0.277, *p*=0.052							-0.574	0.067		
DLCO%pred	*r*=-0.181, *p*=0.209										-0.003	0.214
Age	*r*=0.183, *p*=0.203		0.004	0.246	0.002	0.510	0.001	0.866	0.001	0.730	0.002	0.626
BMI	*r*=0.154, *p*=0.285		0.009	0.440	0.010	0.411	0.013	0.250	0.017	0.159	0.017	0.165
SMOKE		*r*=-0.002, *p*=0.989	-0.002	0.351	-0.002	0.391	-0.002	0.407	-0.001	0.665	-0.014	0.731

Abbreviations: β_1-AAb, β_1-adrenergic receptor autoantibody; RV%pred (residue volume/residue volume predicted percentage); RV/TLC (residue volume/total lung capacity ratio); FEV_1%pred (percent of forced expiratory volume in 1 second); FEV_1/FVC (forced expiratory volume in 1 second /forced vital capacity ration), DLCO%pred (diffusing capacity of the lungs for carbon monoxide/predicted values); BMI, body mass index; SMOKE, smoking history, in pack yeas.
